# Characterization of T Cell Receptors of Th1 Cells Infiltrating Inflamed Skin of a Novel Murine Model of Palladium-Induced Metal Allergy

**DOI:** 10.1371/journal.pone.0076385

**Published:** 2013-10-03

**Authors:** Hiroshi Kobayashi, Kenichi Kumagai, Takanori Eguchi, Hiroaki Shigematsu, Kazutaka Kitaura, Mitsuko Kawano, Tatsuya Horikawa, Satsuki Suzuki, Takaji Matsutani, Kouetsu Ogasawara, Yoshiki Hamada, Ryuji Suzuki

**Affiliations:** 1 Department of Oral and Maxillofacial Surgery, School of Dental Medicine, Tsurumi University, Yokohama, Japan; 2 Department of Rheumatology and Clinical Immunology, Clinical Research Center for Rheumatology and Allergy, Sagamihara National Hospital, National Hospital Organization, Sagamihara, Japan; 3 Department of Oral and Maxillofacial Surgery, Nagano Matsushiro General Hospital, Nagano, Japan; 4 Department of Oral and Maxillofacial Surgery, Toshiba Rinkan Hospital, Sagamihara, Japan; 5 Department of Immunobiology, Institute of Development, Aging and Cancer, Tohoku University, Sendai, Japan; 6 Department of Dermatology, Nishi-Kobe Medical Center, Kobe, Japan; 7 Section of Biological Science, Research Center for Odontology, Nippon Dental University, Tokyo, Japan; University Paris Sud, France

## Abstract

Metal allergy is categorized as a delayed-type hypersensitivity reaction, and is characterized by the recruitment of lymphocytes into sites of allergic inflammation. Because of the unavailability of suitable animal models for metal allergy, the role of T cells in the pathogenesis of metal allergy has not been explored. Thus, we developed a novel mouse model for metal allergy associated with infiltration of T cells by multiple injections of palladium (Pd) plus lipopolysaccharide into the footpad. Using this model, we characterized footpad-infiltrating T cells in terms of phenotypic markers, T cell receptor (TCR) repertoires and cytokine expression. CD3+ CD4+ T cells accumulated in the allergic footpads 7 days after Pd challenge. The expression levels of CD25, interleukin-2, interferon-γ and tumor necrosis factor, but not interleukin-4 and interleukin-5, increased in the footpads after challenge, suggesting CD4+ T helper 1 (Th1) cells locally expanded in response to Pd. Infiltrated T cells in the footpads frequently expressed AV18-1 and BV8-2 T cell receptor (TCR) chains compared with T cells in the lymph nodes and exhibited oligoclonality. T-cell clones identified from Pd-allergic mouse footpads shared identical CDR3 sequences containing AV18-1 and BV8-2. These results suggest that TCR AV18-1 and BV8-2 play dominant and critical parts in the antigen specificity of Pd-specific Th1 cells.

## Introduction

Metal allergy is categorized as a delayed-type hypersensitivity (DTH) reaction, and may be caused by metal ions released from dental materials, jewelry, and coins [1]. Recently, the number of patients with metal allergy has increased because metal is increasingly used for jewelry, surgical instruments, and dental restorations [1]. In addition to nickel (Ni), cobalt (Co) and chromium (Cr), which often induce metal allergy, palladium (Pd) was also reported as a causal metal for allergic contact dermatitis. Dental materials containing Pd have increased because of its resistance to corrosion [2,3]. Therefore, metal allergy caused by Pd ions eluted from dental materials has become a serious problem [4].

Although diagnosis of metal allergy is usually based on patch tests, false positive or negative results are frequently obtained. Furthermore, this procedure carries risks of patient sensitization and specialized training is necessary to interpret the results. The lymphocyte transformation test (LTT) has attracted attention as a potential new method for testing metal allergy. However, the LTT assay in humans can result in non-specific lymphocyte proliferation and false negative results.

Metal allergy is usually associated with the infiltration of lymphocytes into sites of allergic inflammation. Similar to contact hypersensitivity to classical haptens, T cells are essential for mediating metal allergies [5,6]. Metal ions induce the proliferation of human T cells *in vitro* and limited T cell receptor (TCR) repertoires were expressed by human T cells isolated from patients with metal allergy [7-9]. However, the involvement, antigen specificity and diversity of pathogenic T cells in the development of metal allergy remain unclear.

To explore how T cells infiltrating into sites of allergic inflammation contribute to the development of metal allergy, a suitable animal model must be established. On the basis of previous reports [10], we developed a novel murine model of Pd allergy by sensitization twice with Pd plus lipopolysaccharide (LPS) solution into the groin and then three challenges of Pd solution into the footpad. This model represents the DTH response of metal allergy, and allows us to investigate infiltrating T cells in the elicitation phase.

In the present study, we characterized footpad-infiltrating T cells during the elicitation phase of the metal allergy model in terms of phenotypic markers, TCR repertoires and cytokine expression. We found that CD3+ CD4+ T cells infiltrated into the footpads of Pd-induced metal allergy mice. These T cells dominantly used highly oligoclonal TCR repertoires and preferentially expressed T helper type 1 cytokines. This novel murine model is useful for the study of pathogenic roles of T cells in metal allergy and the intriguing results obtained from this study will provide new insights into antigen specificity of TCRs and the role of TCRα chains in Pd-specific T cells.

## Materials and Methods

This study was performed in strict accordance with recommendations in the Guidelines for Care and Use of Laboratory Animals set by the Clinical Research Center for Rheumatology and Allergy, Sagamihara National Hospital, Japan. All animal experiments were performed according to the relevant ethical requirements and with approval from the committees for animal experiments at the Clinical Research Center for Rheumatology and Allergy, Sagamihara National Hospital, Japan (approval number H22-2010-1). All surgery was performed under tribromoethanol anesthesia, and all efforts were made to minimize suffering.

### Animals

BALB/cAJcl mice (5-week-old females) were obtained from CLEA Japan (Tokyo, Japan). Mice were maintained in standard aluminum cages (with a lid made of stainless-steel wire). Food and water were available *ad libitum*.

### Reagents

PdCl_2_ (purity > 95%) was purchased from Wako Pure Chemical Industries (Osaka, Japan). LPS from *Escherichia coli* O55:B5 prepared by phenol–water extraction was purchased from Sigma (St Louis, MO, USA). PdCl_2_ and LPS were dissolved in sterile saline.

### Sensitization, elicitation and measurement of allergic footpad swelling

A total of 125 µl of 10 mM PdCl_2_ with 10 µg/ml LPS in saline was injected twice into the left and right groin of mice at an interval of 7 days *via* the intradermal (id) route (250 µl each injection). At 7 days after sensitization, sensitized mice were challenged for elicitation with 25 µl of 10 mM PdCl_2_ (without LPS) in saline into the left and right footpad by id injection under anesthesia with tribromoethanol. Controls for sensitization and elicitation consisted of injection of saline (25 µl for each footpad). For full elicitation, the challenge was repeated three times at an interval of 14 days. Footpad swelling was measured at the indicated times using a Peacock Dial Thickness Gauge (Ozaki MFG Co. Ltd, Tokyo, Japan). Pd-induced difference in thickness before and after challenge was recorded.

### Immunohistochemical (IHC) analyses

Footpads were obtained from Pd-allergic mice for histology and IHC analyses. Tissue samples were fixed with 4% paraformaldehyde-lysine-periodate overnight at 4°C. After washing with phosphate buffered saline (PBS), tissue samples were incubated in 5% sucrose/PBS for 1 h, 15% sucrose in PBS for 3 h, and 30% sucrose in PBS overnight at 4°C. Tissue samples were embedded in a Tissue Mount (Chiba Medical, Saitama, Japan) and snap-frozen in a mixture of acetone and dry ice. Frozen sections were sliced into 6-µm cryosections and air-dried on poly-L-lysine-coated glass slides. For histological analyses, the cryosections were stained with hematoxylin and eosin (H&E). The cryosections were then stained with anti-mouse CD3ε (145-2C11, Pharmingen, San Diego, CA, USA), CD4 (GK1.5, Pharmingen) and CD8α (53-6.7, Pharmingen) monoclonal antibodies (mAbs). Non-specific binding of mAbs was blocked by incubation of sections with PBS containing 1:20 dilution of normal goat serum or normal rabbit serum, 0.025% Triton X-100 (Wako Pure Chemicals, Osaka, Japan) and 5% BSA (Sigma Aldrich) for 30 min at room temperature (RT). The sections were incubated with primary mAbs for 1 h at RT. After washing three times with PBS for 5 min, intrinsic peroxidase was quenched by 3% H_2_O_2_ in methanol. After soaking the sections in distilled water, they were washed twice. The sections were incubated with a secondary Ab (biotinylated goat anti-hamster IgG antibody for CD3ε detection or biotinylated rabbit anti-rat IgG antibody for CD4 and CD8α detection) for 1 h at RT. After washing three times, the sections were incubated with the Vectastain ABC Reagent (Vector Laboratories, Burlingame, CA, USA) for 30 min at RT, followed by 3,3'-diaminobenzidine (DAB) staining (0.06% DAB and 0.03% H_2_O_2_ in 0.1 M Tris-HCl buffer pH 7.6, Wako Pure Chemicals). Finally, the tissue sections were stained with hematoxylin to visualize cell nuclei.

For image cytometry, digital images of immunohistochemistry were analyzed using Histoquest software (TissueGnostics, Vienna, Austria). Briefly, nucleated cells were detected in the hematoxylin channel and a nucleus detection algorithm was determined. Then, mAb-positive cells were detected by DAB signals in the nucleated cells. DAB intensity was plotted against the hematoxylin intensity to create a scattergram. The cut-off threshold was uniform for all images.

### Isolation of total RNA from tissues

Fresh footpads and spleens were obtained from mice and immediately soaked in RNAlater RNA Stabilization Reagent (Qiagen, Hilden, Germany). Total RNAs from footpads and spleens were extracted using the RNeasy Lipid Tissue Mini Kit (Qiagen) according to the manufacturer’s instructions.

### Quantitative PCR (qPCR)

The expression levels of mRNA for immune response-related genes including T cell-related CD antigens, cytokines, and cytotoxic granules, were measured by qPCR method using the Bio-Rad CFX96 system (Bio-Rad, Hercules, CA, USA). Specific primers for GAPDH, CD3, CD4, CD8, interferon (IFN)-γ, tumor necrosis factor (TNF)-α, interleukin (IL)-2, IL-4, IL-5, IL-10, T-bet, GATA3, CD25 (IL-2Rα), perforin, granzyme A, granzyme B, and Fas ligand (FasL) were described previously [11]. Histamine-forming enzyme, histidine decarboxylase (HDC)-specific primers were purchased from Takara Bio (Otsu, Japan). GAPDH gene expression was used as internal control. The expression levels of each target gene were normalized to GAPDH expression. 500ng of freshly isolated total RNA from the footpads of mice was converted to cDNA (final 10µl) using PrimeScript™ RT Reagent Kit (Takara Bio, Otsu, Japan) according to the manufacturer’s instructions. The PCR reaction mixture consisted of 5 µl of SsoFast™ EvaGreen^®^ Supermix (Bio-Rad), 3.5 µl of RNase/DNase-free water, 0.5 µl of 5 µM primer mix, and 1 µl of cDNA in a total volume of 10 µl. Cycling conditions were as follows: 30 s at 95°C followed by 45 rounds of 1 s at 95°C and 5 s at 60°C. Melting curve analyses were undertaken from 65°C to 95°C to confirm homogeneity of PCR products.

### TCR repertoire analysis

TCR repertoire analysis was performed using samples from Pd -challenged mice and control mice (n = 10) by an adaptor ligation-mediated PCR and microplate hybridization assay method [12-14]. Briefly, total RNA was converted to double-stranded cDNA using Superscript cDNA synthesis kit (Invitrogen, Carlsbad, CA) according to the manufacturer’s instructions, except that a specific primer (BSL-18E) was used [14]. The P10EA/P20EA adaptor was ligated to the 5’ end of the cDNA and was cut with *Sph*I. PCR was performed using either TCR α-chain constant region-specific (MCA1) or TCR β-chain constant region-specific primers (MCB1) and P20EA. The second PCR was performed with either MCA2 or MCB2 and P20EA primers. The third PCR was performed using both P20EA and either 5’-biotinylated MCA3 or MCB3 primers for biotinylation of PCR products. Ten pmol of amino-modified oligonucleotides specific for the TRAV and TRBV segments were immobilized onto carboxylate-modified 96-well microplates with water-soluble carbodiimide. Prehybridization and hybridization were performed in GMCF buffer (0.5 M Na _2_HPO; pH 7.0, 1 mM EDTA, 7% SDS, 1% BSA and 7.5% formamide) at 47°C for 30min. One hundred microliters of the denatured 5’-biotinylated PCR products were mixed with the equivalent volume of 0.4 N NaOH/10 mM EDTA, and the mixture was added to 10 mL of GMCF buffer. One hundred microliters of hybridization solution was used in each well of the microplate containing immobilized oligonucleotide probes specific for V segments. After hybridization for 2 h, the wells were washed 4 times with washing buffer (2 × SSC, 0.1% SDS) at room temperature. The plate was incubated at 37°C for 10 min for stringency washing. After washing 4 times with the same washing buffer, 200 µL of TB-TBS buffer (10 mM Tris-HCl, 0.5 M NaCl, pH 7.4, 0.5% Tween-20 and 0.5% blocking reagent; Roche Diagnostics) was added to block nonspecific binding. Next, 100 µL of 1:2,000-diluted alkaline phosphatase-conjugated streptavidin in TB-TBS was added, and the sample was incubated at 37°C for 30 min. The plates were washed 6 times in T-TBS (10 mM Tris-HCl, 0.5 M NaCl, pH 7.4, 0.5% Tween 20). For color development, 100 µL of substrate solution (4 mg/mL p-nitrophenylphosphate (Sigma Aldrich) in 10% diethanolamine, pH 9.8) was added, and absorbance was determined at 405 nm. The ratio of the hybridization intensity of each TCRV-specific probe to that of a TRC-specific probe (V/C value) was determined using the TCR cDNA concentrated samples that contained the corresponding V segment and the universal C segment, respectively. Absorbance obtained with each TCRV-specific probe was divided by the corresponding V/C value. The relative frequency was calculated using the corrected absorbency using the following formula: relative frequency (%) = (corrected absorbance of TCRV-specific probe/the sum of corrected absorbencies of TCRV-specific probes) × 100.

### T cell clonality analysis with CDR3 size spectratyping

The level of T cell clonality was evaluated in samples from Pd-challenged mice (n=5) and control mice (n=5) using a CDR3 size spectratyping method [11,15]. PCR was performed for 30 cycles in a 20 µL volume under the same conditions as described above. The PCR mixture consisted of 1 µL of 1:20 or 1:50 diluted second PCR product, 0.1 µM of 5’-Cy5 MCA3/ MCB3 primer and 0.1 µM primer specific for each variable segment. Two microliters of 1:20 diluted PCR product was analyzed with the CEQ8000 Genetic Analysis System. Spleens of BALB/cAJcl mice were used as controls showing a Gaussian distribution pattern with multiple peaks.

### Determination of CDR3 nucleotide sequences

PCR was performed with 1 µL of 1:20 diluted second PCR product, using a forward primer specific for the variable region and a reverse primer specific for the constant region (MCA4 or MCB4) under the conditions described above. The primers used in this study were as follows: AV18-1: 5’-AGACTCCCAGCCCAGTGACT-3’, BV8-2: 5’-GGCTACCCCCTCTCAGACAT-3’. PCR products eluted from the agarose gel were cloned into the pGEM-T Easy Vector (Promega, Madison, WI, USA). The recombinant plasmid DNA was transfected into DH5α competent cells. Sequence reactions were performed with the GenomeLab DTCS Quick Start Kit (Beckman Coulter) and analyzed by the CEQ8000 Genetic Analysis System. A total of 505 clones from the footpads of Pd- induced allergy mouse were examined in this experiment (n=5).

### Establishment of Pd-allergic specific T cell clones

Pd-allergic BALB/c mice (n=8) were killed under general anesthesia 7 days after challenge and their footpads were removed. The footpads were kept on ice in RPMI 1640 medium containing 10% FCS. The footpad specimens were finely minced into small pieces, soaked in a solution of enzyme cocktails containing 0.1% type iv collagenase (Sigma, St. Louis, MO), 0.1% hyaluronidase (Sigma), and 0.01% DNase (Sigma), and incubated for 2 hours at 37°C in a shaking water bath. After removal of debris by pressing through a 100-mm mesh tissue strainer (BD Pharmingen, San Jose, CA), the obtained cells were suspended in RPMI 1640 medium (Gibco, Grand Island, NY) and washed twice. Homogenates were centrifuged at 400*g* for 10 min at 22°C, and cell pellets were resuspended in 5 ml RPMI 1640 and layered over Lympholyte-Separation Medium (M-SMF, JIMRO, Tokyo) before centrifugation at 1500*g* for 20 min at 22°C. Next, isolated lymphocytes were cultured in 1 ml AIM-V medium (Gibco) containing 10% FCS, 100 U of recombinant IL-2 (Shionogi & Co., LTD, Japan) per ml, and 2.0 µg/ml concanavalin A (ConA, Sigma) in 24-well plates (Corning Costar, Corning, NY) at 37°C for 7 days.

Cells were incubated with appropriately diluted, fluorescein isothiocyanate (FITC)-labeled anti-mouse TCR BV8 (F23.1, BD Pharmingen) for 15 min at 4°C and washed with BSA-containing PBS. Stained cells were collected cell sorting using the FACSAria iii system (Becton Dickinson, Franklin Lakes, NJ).

Collected TCR BV8-positive T cells were cultured at 0.5-1.0 cells/well with feeder cells (BALB/c splenocytes treated with 0.025 mg/ml of mitomycin C, 5×10^6^ cells/well) in AIM-V medium supplemented with L-glutamine, penicillin-streptomycin (100 U/ml), 10% FCS, 100 U of IL-2, and 0.1-1.0 µM PdCl_2_ in 96 well U-bottom microtiter plates (Coster). After the second week, five T cell clones (Clones A08, D03, D07, E02, and G02) were established by the limiting dilution method as described previously [16].

### IFN-γ release assay and CDR3 sequences analyses

Peritoneal exudate cells (PECs) were collected from BALB/c mice (n=3), and adjusted to 5.0×10^4^ cells/ml, and 100 µl PECs were dispensed into the Nunc, Flat-bottom 96-wells (Thermo Scientific, Waltham, MA). Subsequently, the T cell clones were plated at 1.0×10^5^ cells/well, and cultured in in AIM-V medium supplemented with L-glutamine, penicillin-streptomycin (100 U/ml), and 10% FCS at 37°C in 5% CO_2_ for 24 h. For activation, PdCl_2_ at 0, 0.1, 0.5, 1 and 10 mM was added. Culture supernatant fluids were collected, and levels of IFN-γ were measured by enzyme-linked immunosorbent assay (ELISA) (R&D Systems, Minneapolis, MN). Each T cell clone was cultured further for 3 days, and used for CDR3 sequence analyses.

### Statistical analysis

Differences were statistically analyzed using the Student’s unpaired *t*-test using StatView 5.0 for Windows (SAS Institute Inc., Cary, NC). A *P* value < 0.05 was determined to be statistically significant.

## Results

### Footpad swelling in Pd-induced allergic mice

**Figure 1:**
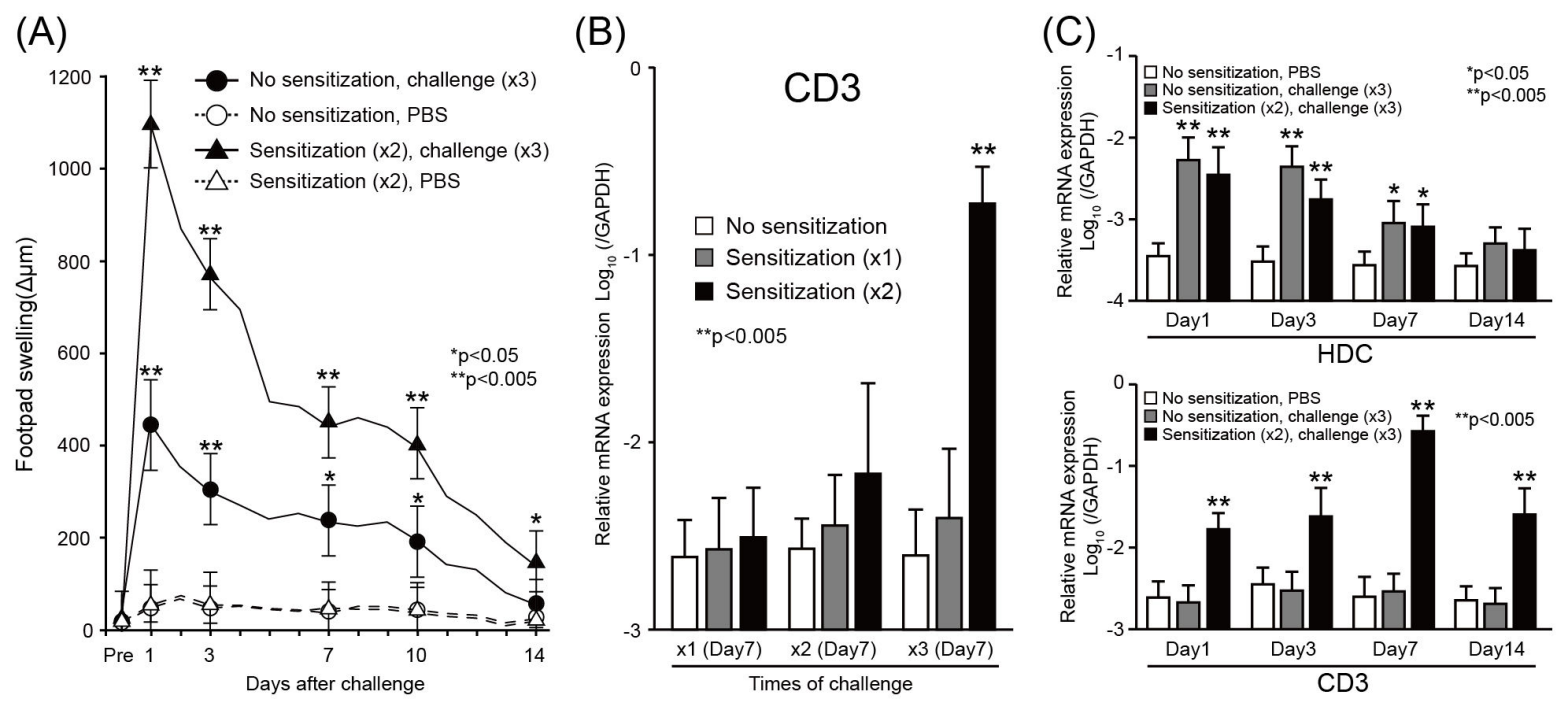
Footpad swelling and expression of T-cell markers in Pd-induced allergic mice. (A) Time course of footpad swelling under different conditions of sensitization and challenge. BALB/c mice were sensitized twice with 125µl of 10 mM Pd and 10 µg/ml LPS and then challenged three times with 25 µl of 10 mM PdCl_2_ (closed triangle) or PBS (open triangle) (n=7). In addition, mice were challenged three times with Pd (closed circle) or PBS (open triangle) without sensitization (n=10). Changes of footpad thickness were measured before and 1, 3, 7, 10 and 14 days after the last challenge of Pd. Data were indicated mean ± standard deviation (SD). Statistical significance was tested by unpaired Student’s *t*-test. (B) Expression levels of CD3ε mRNA were dependent upon sensitization condition. Mice were treated with different sensitization and challenge conditions (n=7). CD3ε expression was measured by qPCR 7 days after the last challenge. Bars and error bars indicate mean ± standard deviation. Statistical significance was only detected in mice administered two sensitizations and three challenges. (C) Time course of gene expression for histidine decarboxylase (HDC) and CD3ε in Pd-induced allergy mice. Expression of HDC and CD3ε were measured by qPCR at different time points after the last challenge. Data indicates mean ± SD (n=10). Statistical significance was tested by unpaired Student *t*-test (*p<0.05, **p<0.005).

To establish a new murine model for metal allergy, we evaluated the conditions required to induce metal allergy in BALB/c mice by multiple injection of Pd and LPS. Mice were sensitized twice with Pd and LPS, and then challenged three times with Pd. Footpad swelling was measured every day after the last challenge of Pd (Figure 1A). Sensitization did not affect footpad swelling in PBS-challenged mice. In contrast, three times of challenges of Pd induced swelling in non-sensitized miceremarkably enhanced swelling in Pd-sensitized mice. These results indicated that sensitization by Pd and LPS significantly enhanced footpad swelling in response to secondary injection of Pd. The peak of footpad swelling was observed 24 h after the last challenge of Pd in all groups. The swelling continued up to 10 days and then returned to basal levels 14 days after the last challenge.

### Expression of T-cell markers in the footpads of Pd-induced allergic mice

To verify the presence of footpad-infiltrating T cells, the mRNA expression level of CD3ε was measured by qPCR. First, we compared the expression levels of CD3ε among groups of mice with different numbers of Pd sensitization and challenge to determine the conditions that effectively induced T cell infiltration into the site of allergic inflammation (Figure 1B). Three times of Pd challenge increased CD3ε expression in the twice-sensitized mice, but not in the once- and unsensitized mice. Then, we examined whether CD3ε expression increased time-dependently in the twice-sensitized mice. The CD3ε expression significantly increased at day 1 and reached its highest at day 7 after challenge (Figure 1C). To determine whether histamine, which is reported to be related to metal allergy, also contribute to induction of Pd allergy, histidine decarboxylase (HDC) expression levels were evaluated by qPCR. Significant increases in the expression levels of HDC gene, which is related to histamine production, were observed early in both the non- and the twice-sensitized mice. These results suggest that a combination of twice sensitizations of Pd and LPS and three times of Pd challenge effectively induced infiltration of T cells in the inflamed skin of Pd-induced allergy mice independent of histamine production.

### IHC analyses in footpads of Pd-induced allergy mice

**Figure 2:**
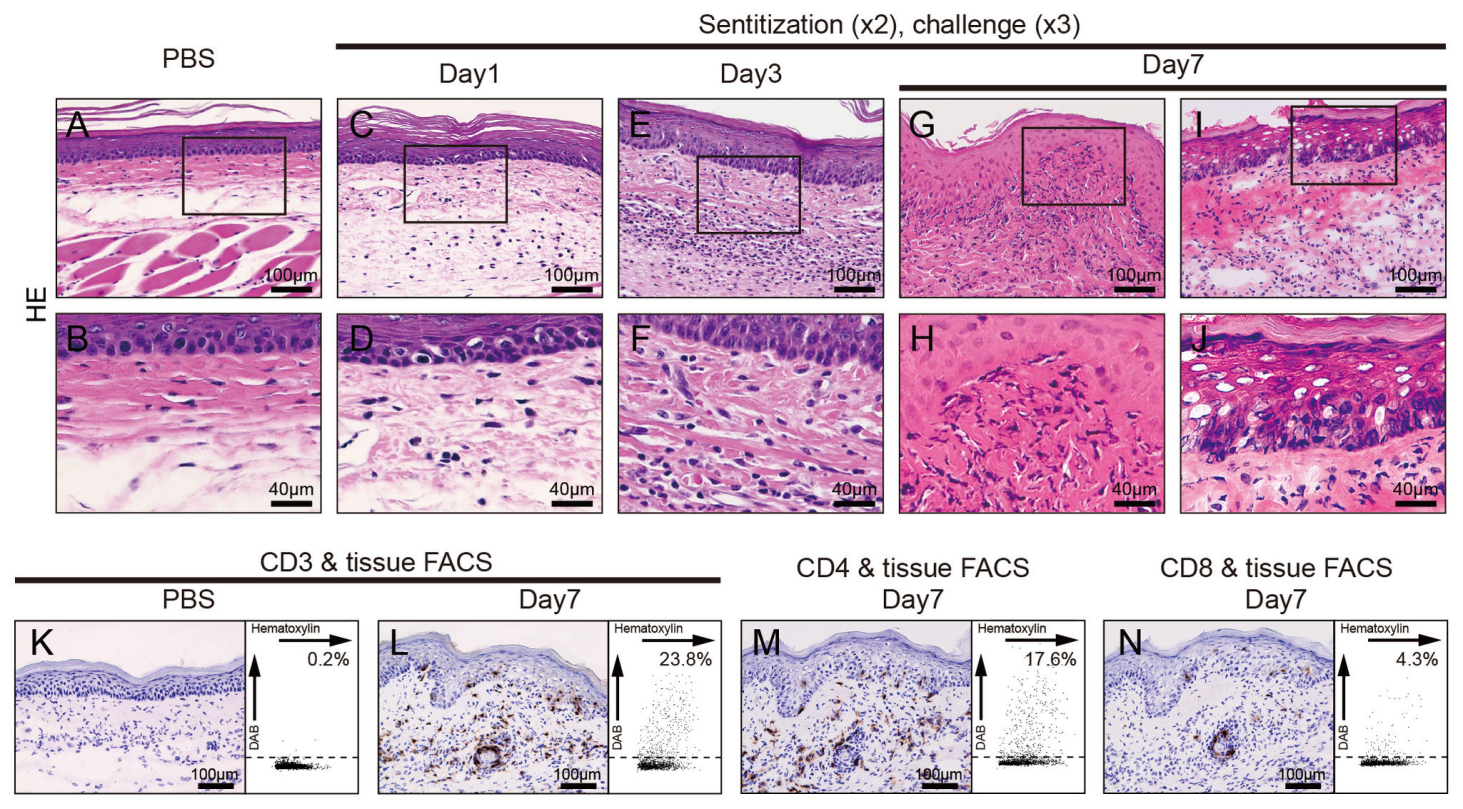
Immunohistochemical analyses of footpads from Pd-induced allergic mice. Histopathology and immunohistochemical (IHC) analyses were undertaken to identify CD3+, CD4+ and CD8+ cells in footpad tissues. Frozen sections of the footpad tissues were prepared from saline-injected mice or Pd-induced allergy mice (two sensitizations–three challenges) at 7 days after the last challenge. Sections were stained with H&E (C–J), anti-mouse CD3ε (K and L), anti-CD4 (M) and anti-CD8 mAbs (N) and visualized by DAB. Representative photomicrographs are shown. Liquefaction degeneration of basal epithelial layer and infiltration of inflammatory cells into the epithelial basal layer and dermis were observed in Pd-induced allergy mice (G-J), but not control mice (A and B). For IHC analyses, epidermotropism of CD3+ cells was present in the epithelial basal layer and dermis of the Pd-induce allergy mice (L), but not control mice (K). CD4^+^ T cells were abundant (M) while a small number of CD8+ T cells existed only around blood vessels (N). Representative IHC images were analyzed by HistoQuest software and the data was shown as scatter grams on the right.

To verify whether T cells infiltrated into the site of inflamed skin, we analyzed the footpad skin of Pd-induced allergy mice and control mice at 7 days by IHC. H&E staining showed dense mononuclear cells infiltrate in the epithelial basal layer and upper dermis, and liquefaction degeneration of the epithelial basal layer (Figure 2G-J). IHC staining showed that CD3+ T cells were also present in the epithelial basal layer and the upper dermis, but not in control mice (Figure 2L, K). Furthermore, CD4+ T cells had preferentially infiltrated into the skin tissues, while perivascular infiltration of small numbers of CD8+ T cells was observed (Figure 2M, N). To evaluate quantitatively the number of T cell infiltrates in the dermal skin, we performed IHC using image cytometry. Analysis indicated that CD3+ T cell infiltrates were present at day 1 (5.3%, data not shown) and the percentage of CD3+ T cells increased until day 7 (23.8%, Figure 2L). Interestingly, the percentage of CD4+ T cell infiltrates was 4-fold higher than for CD8+ T cells (17.6% vs. 4.8%) (Figure 2 M, N). These results indicated that CD4+ T cells rather than CD8+ T cells preferentially infiltrated into in response to Pd in the skin of Pd-induced allergy mice.

### Levels of cytokines, chemokines, chemokine receptors and cytotoxic granules in footpads

**Figure 3:**
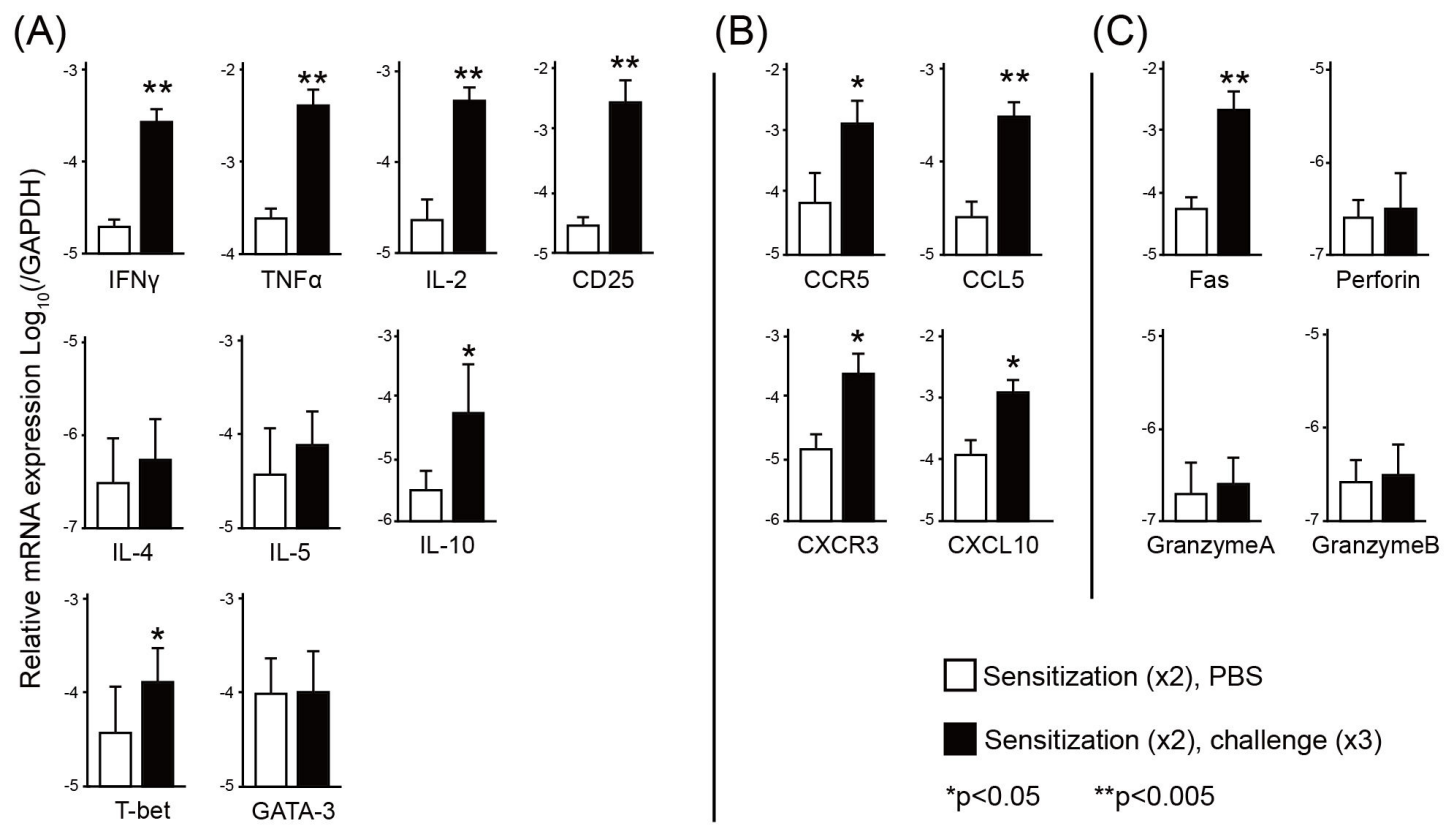
Quantification of T cell related markers and cytokines by qPCR. The mRNA expression of CD25, cytokines (IFN-γ, TNF-α, IL-2, IL-4, IL-5, T-bet, GATA3, and IL-10), chemokines and chemokine receptors (CCR5, CCL5, CXCR3, and CXCL10), and cytotoxic molecules (FasL, perforin, granzyme A and granzyme B) are shown (A-C). RNA was extracted from footpads of Pd-induced allergy mice (two sensitizations/three challenges, closed bars) and control mice (two sensitizations, open bars). GAPDH gene expression was used as an internal control. Vertical bars and error bars indicate the mean values and standard deviation (n=10). Significant differences compared with PBS-injected footpads were analyzed by unpaired Student *t*-test (*p<0.05, **p<0.005). All experiments were independently performed twice.

Cytokine expression was measured from the footpads of Pd-challenged mice by qPCR. Significant increases of the expression levels of IFN-γ, TNF-α, IL-2, T-bet (Th1 type cytokines) and CD25, were found in the footpads of Pd-challenged mice compared with control mice (Figure 3A). In contrast, the expression levels of Th2 type cytokines, IL-4, IL-5, and GATA-3 were not significantly increased except for IL-10 (Figure 3B). The expression levels of chemokines (CCL5 and CXCL10) and chemokine receptors (CCR5 and CXCR3) were considerably increased in the footpads after the last challenge of Pd (Figure 3C). Furthermore, we examined the levels of cytotoxic molecules to determine the apoptotic environment in the skin. Levels of Fas was significantly higher at day 7 in the footpads of twice-sensitized–three times-challenged mice compared with those of control mice (Figure 3D). These results indicated that the levels of Th1-type cytokines, chemokines, and cytotoxic molecule were positively associated with the progression of Pd-induced metal allergy.

### TCR repertoires in skin of Pd-induced allergy mice

**Figure 4:**
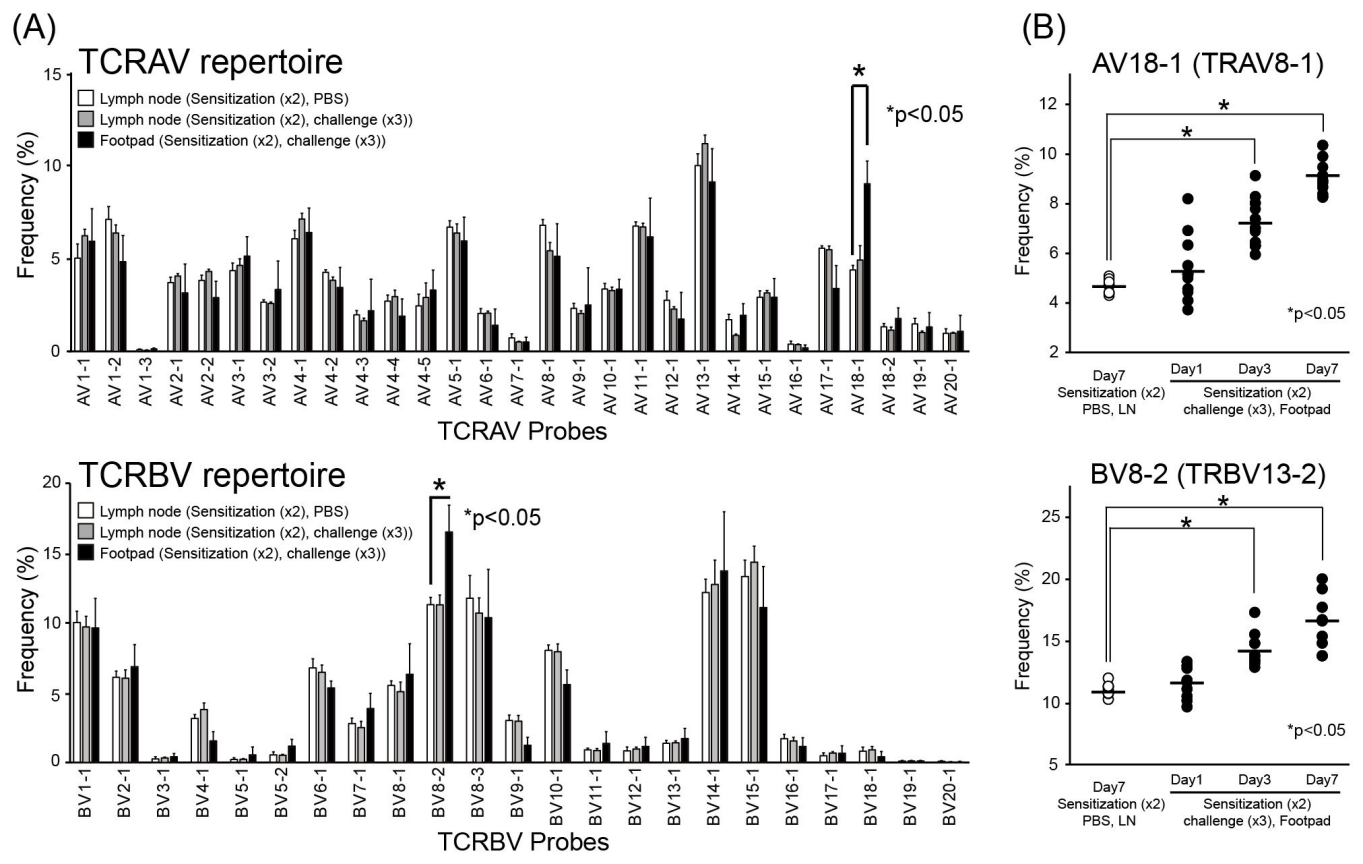
TCR repertoire analyses in Pd-induced allergic mice. (A) TRAV and TRBV repertoires were analyzed from footpads of Pd-induced allergy mice (two sensitizations–three challenges) by microplate hybridization assay (n=10). Lymph nodes from corresponding Pd-injected mice and PBS-injected mice were used as controls. The vertical bars and error bars indicate the mean values and standard deviations, respectively. At 7 days after the last challenge, percentage frequencies of the expression levels of AV18-1 and BV8-2 were significantly higher in footpads of Pd-induced allergy mice compared with lymph nodes of control mice (*p<0.05, unpaired Student *t*-test). (B) Increases in the frequencies of AV18-1 and BV8-2 after the last challenge of Pd. TRAV and TRBV repertoires were analyzed from RNA samples from footpads of Pd-induced allergy mice obtained at day 1, 3 and 7. The frequencies of AV18-1 and BV8-2 in Pd-induced allergy mice significantly increased at day 3 and 7 compared with control mice (*p<0.05, unpaired Student *t*-test). All experiments were performed in triplicate.

**Figure 5:**
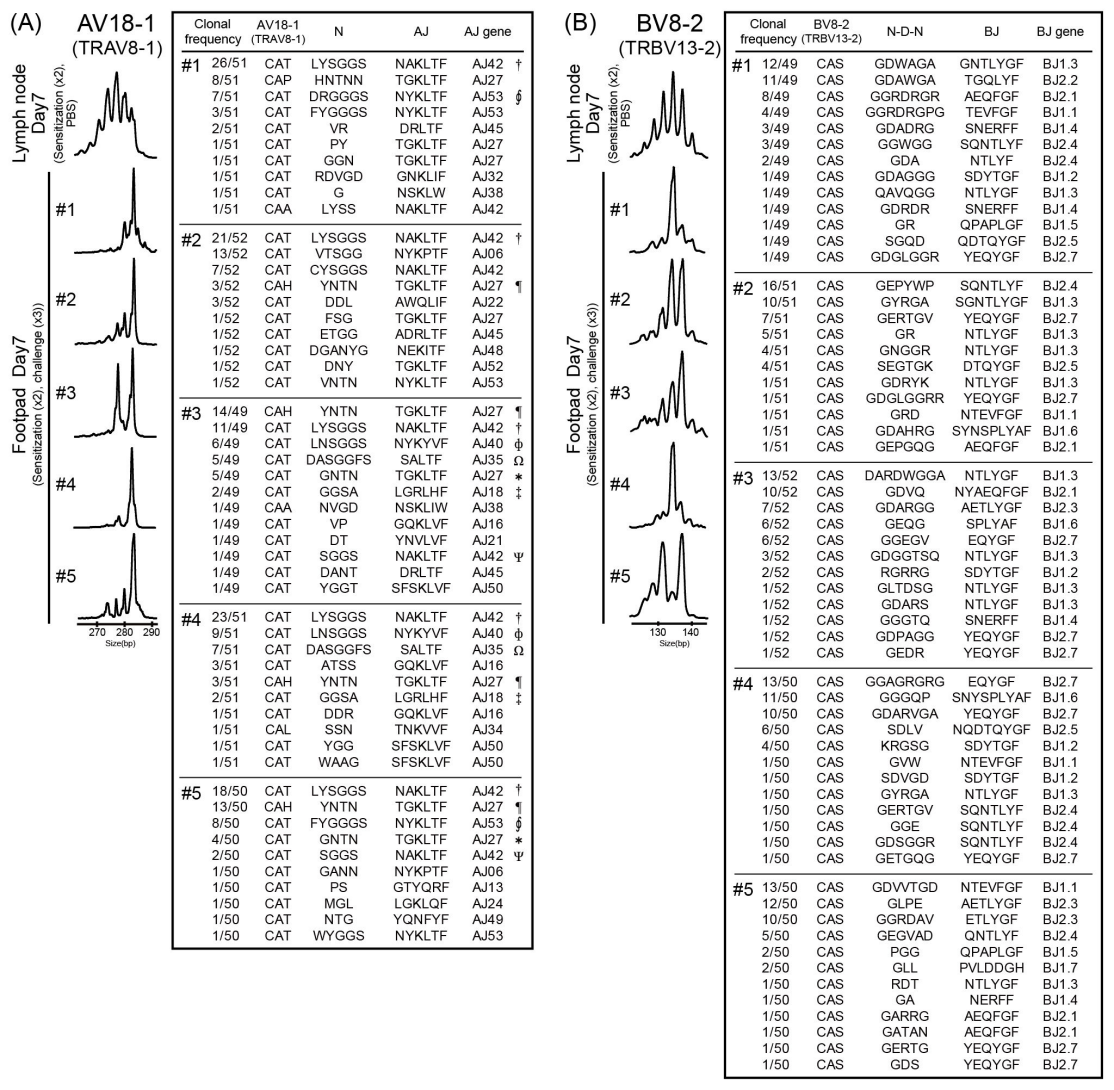
T cell clonality and characteristics of CDR3 sequences in TCR clonotypes bearing AV18-1 and BV8-2. T cell clonality was analyzed by CDR3 size spectratyping and nucleotide acid sequences were determined in TCR clonotypes bearing AV18-1 and BV8-2. RNA was extracted from footpads of Pd-induced allergy mice at 7 days (n=5) and nucleotide acid sequences of CDR3 were determined in 49-52 cDNA clones per mouse. Representative profiles of CDR3 size spectratyping and deduced amino acid sequences of the CDR3 bearing AV18-1 (A) and BV8-2 (B) are shown. (A) For AV18-1, a polyclonal peak pattern (multiple peaks with Gaussian distribution) was obtained from lymph nodes of control mice. In contrast, Pd-induce allergy mice exhibited oligoclonal peak patterns with a dominant peak at the same position among five mice. Identical CDR3 sequences (†, ¶, *, Ω, Φ, ‡, Ψ) were obtained from the footpads of different mice. (B) For BV8-2, Pd-induced allergy mice showed oligoclonal peak patterns but they were different among the mice. CDR3 sequence analysis also showed multiple predominant clones presented in each mouse but they did not share CDR3 sequences among different mice. All experiments were performed in triplicate.

To examine whether TCR repertoires are skewed at the sites of inflamed skin, we analyzed TCR α-chain variable (TRAV) and TCR β-chain variable (TRBV) repertoires in Pd-induced allergic BALB/c mice. The percentage of AV18-1 (IMGT gene name: TRAV8-1) and BV8-2 (IMGT gene name: TRBV13-2) usage was significantly higher in the footpads of Pd-induced allergic mice compared with lymph nodes of the corresponding mice (Figure 4A, Table 1). There were no significant differences in the percentage usage of other TRAVs and TRBVs in Pd-induced mice between the footpads and the lymph nodes (n=10) and in the lymph nodes between Pd-induced mice and control mice (n=10). The expression of AV18-1 and BV8-2 increased gradually in the footpads during 7 days after last challenge (Figure 4B), indicating that their increased expression correlated with the accumulation of CD4+ T cells. Furthermore, T cell clonality was examined by CDR3 size spectratyping. For AV18-1 and BV8-2, polyclonal peak patterns were detected in the lymph nodes of Pd-induced allergic mice and control mice, while a few oligoclonal peak patterns were detected in the footpad of Pd-induced allergic mice (Figure 5). A dominant peak within the multiple peaks for AV18-1 appeared to be identical in size among individual mice, whereas an oligoclonal peak pattern was detected in BV8-2. Sequencing analyses showed that identical TCR clonotypes bearing AV18-1 were obtained from the footpads of different mice while diverse TCR clonotypes with BV8-2 were obtained from the footpads of all mice. These results indicated that T cells bearing AV18-1 and BV8-2 are responsible for metal allergy. Furthermore, certain T cells bearing AV18-1 and BV8-2 were expanded and continuously present for >1 weeks in the inflamed skin.

**Table 1:** Correspondence between probe names and IMGT gene names.

TCRAV			TCRBV	
Probe	IMGT gene		Probe	IMGT gene
AV 1-1	TRAV7-2,3,4,6		BV 1-1	TRBV5
AV 1-2	TRAV7-5		BV 2-1	TRBV1
AV 1-3	TRAV7-1		BV 3-1	TRBV26
AV 2-1	TRAV14-1,2		BV 4-1	TRBV2
AV 2-2	TRAV14-3		BV 5-1	TRBV12-2
AV 3-1	TRAV9-2,3,4		BV 5-2	TRBV12-1
AV 3-2	TRAV9-1		BV 6-1	TRBV19
AV 4-1	TRAV6-6		BV 7-1	TRBV29
AV 4-2	TRAV6-7		BV 8-1	TRBV13-3
AV 4-3	TRAV6-1,2,3,5		BV 8-2	TRBV13-2
AV 4-4	TRAV6-5		BV 8-3	TRBV13-1
AV 4-5	TRAV6-4		BV 9-1	TRBV17
AV 5-1	TRAV3-3		BV10-1	TRBV4
AV 6-1	TRAV21		BV11-1	TRBV16
AV 7-1	TRAV15-1		BV12-1	TRBV15
AV 8-1	TRAV12-1		BV13-1	TRBV14
AV 9-1	TRAV17		BV14-1	TRBV31
AV10-1	TRAV13-1,2,3,4,5		BV15-1	TRBV20
AV11-1	TRAV4-2,3,4		BV16-1	TRBV3
AV12-1	TRAV2		BV17-1	TRBV24
AV13-1	TRAV5-4		BV18-1	TRBV30
AV14-1	TRAV11		BV19-1	TRBV21
AV15-1	TRAV10		BV20-1	TRBV23
AV16-1	TRAV20P			
AV17-1	TRAV16			
AV18-1	TRAV8-1			
AV18-2	TRAV8-2			
AV19-1	TRAV1			
AV20-1	TRAV19			

### Comparison of footpad-infiltrating T cells *in vivo* and *in vitro*

**Figure 6:**
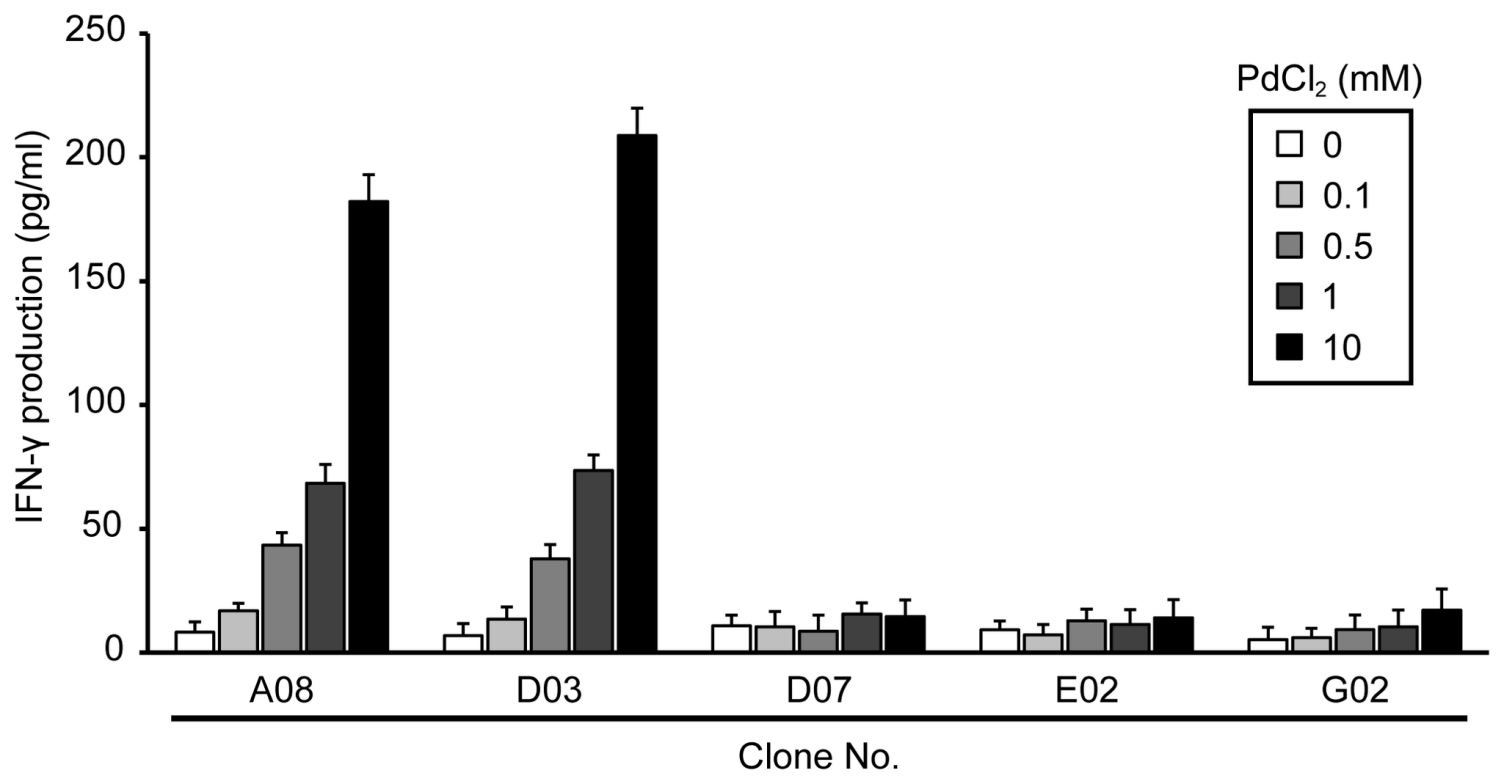
Production of IFN-γ by T cell clones stimulated with Pd. Levels of IFN-γ were measured with culture supernatants from T cell clones stimulated with various concentrations of PdCl_2_ (0.1-10 mM). Bars and error bars represent mean and standard deviation of IFN-γ (pg/mL). High levels of IFN-γ were detected in the culture supernatants of T cell clones A08 as well as D03 at 10 mM PdCl_2_ solution and the production was increased in a dose-dependent manner. On the other hands, the levels of IFN-γ at 0.1, 0.5, 1 and 10 mM Pd were not significantly different from control (0 Pd). Experiments were repeated at least thrice.

The specificity of T cells infiltrating Pd-allergic mouse footpads *in vivo* and *in vitro* was compared. T cell clones isolated from Pd-allergic mouse footpads were cultured for 24 h with Pd Cl_2_ in the presence of PECs. Levels of IFN-γ in the culture supernatants were measured by ELISA. In addition, CDR3 sequences of TCR AV and BV in each T cell clone were examined. T cell clones A08 and D03 isolated from Pd-allergic mouse footpads produced high levels of IFN-γ in response to Pd in a dose dependent manner while no responses were found in the other three clones (Figure 6). Interestingly, T cell clones A08 and D03 exhibited identical CDR3 sequences in AV18-1 (CAT-LYSGGS-NAKLTF) with BV8-2, which was also detected *in vivo* (Table 2, Figure 5A). In contrast, the others have diverse AVs other than AV18-1 and either BV8-1 or 8-2. These results showed that Pd-specific Th1 cells utilized TCR consisting of AV18-1 and BV8-2 at the single cell levels. 

**Table 2:** Amino-acid sequences of CDR3 regions of Pd-induced T cell clones.

	TCRAV						TCRBV				
	V	CDR3			J		V	CDR3			J
Clone	segment	V	N	J	segment		segment	V	N-D-N	J	segment
A08	18-1	CAT	LYSGGS	NAKLTF	42		8-2	CAS	GDAWGAA	NTLYGF	1.3
D03	18-1	CAT	LYSGGS	NAKLTF	42		8-2	CAS	GGWRAG	GNTLYGF	1.3
D07	2-1	CAAS	GE	SGTYQRF	13		8-1	CAS	GEAVGDD	QYGF	2.7
E02	13-1	CAA	VELE	ADRLTF	45		8-1	CAS	GLVTEG	ETLYGF	2.3
G02	1-1	CAVS	AGE	NTGKLTF	27		8-2	CAS	GDGGEV	TEVFGF	1.1

## Discussion

Metal allergy is a DTH response, involving the recruitment of lymphocytes and inflammatory cells including T cells, dendritic cells, granulocytes and macrophages to the site of allergic inflammation. The histopathological features of DTH response are spongiosis, and liquefaction degeneration of epidermis, caused by intercellular edema and lymphocytic infiltration in the epidermis [17]. However, the mechanism by which metal induces allergic inflammation associated with infiltration of lymphocytes is poorly understood. In the present study, we established a novel murine model for Pd-induced allergy in which CD3+ T cells infiltrated the inflamed area. Histological sections showed apparent spongiosis changes with the recruitment of T cells in the inflamed footpad skins. Furthermore, the common morphological features of DTH, such as epidermis tropism of mononuclear cells, and infiltration of inflammatory cells into dermis were also identified. Infiltrating T cells expressed CD4+ and Th1 type cytokines, and interestingly they used a specific TCR repertoire expressing AV18-1 and BV8-2. This study provides evidence for the first time that specific TCR-expressing T cells expanded in response to Pd in inflamed skin.

Previous studies have shown that T cells are largely responsible for the development of metal allergy in mice and humans [18]. Our results indicated that Th1 type cytokines were preferentially produced in response to Pd. The expression of IFN-γ, TNF-α and IL-2 were markedly detected on day 7, suggesting that chemokines and cytokines released by antigen-stimulated Th1 cells may contribute to the pathogenesis of the DTH response. Apart from T cells, mast cells, NK cells and granulocytes have been reported to be involved in metal-induced allergy inflammation in other animal models [10,19,20]. Naïve CD4+ T cells primed with Pd differentiate into memory/effector Th1 cells on day 7. They specifically recognize antigens at the local site to produce cytokines and chemokines, resulting in recruitment of macrophages to inflamed sites. IFN-γ and TNF-α produced by the Th1 cells activate macrophages and occasionally kill macrophages and other sensitive cells by Fas ligand signaling. It is of interest that IL-10 was abundantly expressed in the footpads on day 7. IL-10 controls host immune responses by suppressing Th1 responses [21], and controlling the balance of Th1/Th2 cytokines.

Footpad thickness and the expression of HDC reached its maximum at day 1 while the highest expression of CD3 was observed at day 7. This result was consistent with a previous report of human patch testing [22]. Metal allergy can be elicited by either CD4+ or CD8+ T cells dependent on the pathway by which antigen is processed. We found CD4+ T cells accumulated in large numbers in the inflamed skin, suggesting that infiltrating T cells react to self-antigens presented by major histocompatibility antigen (MHC) class II, however, the precise role of HDC requires further investigation.

Metal ions can easily penetrate intact skin, especially if they cause itching that leads to scratching. Metal ions can act as haptens that covalently bind to proteins or peptides. Metal ions form geometrically highly defined, but reversible, coordination complexes with self-proteins, creating metal ion-protein complexes that can be processed by antigen-presenting cells and presented by MHC molecules [23,24]. Thus, Pd-specific TCRs can recognize a hapten-modified peptide presented by MHC and therefore, similar to conventional peptide antigen, both the CDR3α and 3β may be essential for antigen recognition. However, we observed diverse CDR3β sequences in BV8-2, suggesting that the Pd-specific TCR is unlikely to react with the peptide-MHC complex (pMHC) in a classical hapten-like manner or, alternatively, that it has a different role in antigen recognition between TCR α-chain and β-chains.

CDR3 sequence analyses revealed interesting results with regard to Ag specificity. TCR clonotypes observed in Pd-induced allergic mice showed preferential use of AJ segments (AJ18, 27, 35, 40, 42, and 53 for AV18-1). In contrast to the very restricted TRAV repertoire, footpad-infiltrating T cells exhibited a relatively broad TRBV repertoire. In BV8-2, CDR3 sequences varied considerably among individual mice. It has been proposed that Ni ions function as non-classical haptens, which may activate T cells by crosslinking their receptor to MHC molecules, independent of the nature of the associated peptide [25,26]. Therefore, CDR3α may be essential for intramolecular bridges by Ni ions between TCR and MHC and the subsequent activation of Ni-specific T cells [21]. Interestingly, we also observed high sequence identity of CDR3α in AV18-1, suggesting CDR3α is essential for Pd-specific T cell responses in a peptide-independent manner. Notably, Pd-specific T cells used BV8-2 with diverse CDR3β. This may suggest that germline CDR1β and/or CDR2β within BV8-2 also contribute to the recognition of the Pd-modified antigen peptide.

We have also established Pd-allergic specific T-cell clones, and analyzed TCR usage by these clones. Two of the T-cell clones with high levels of IFN-γproduction showed identical usage of the CDR3 sequence (CAT-LYSGGS-NAKLTF) in AV18-1 with BV8-2, which was also found in each individual mouse *in vivo*. These results strongly suggest that TCR consisting of AV18-1 with a particular CDR3 sequence (CAT-LYSGGS-NAKLTF) and BV8-2 with diverse CDR3 probably has the potential to specifically recognize Ag in Pd allergy.

In contrast with a predominant role of the TCR α chain in the Pd-specific TCR, the TCR β-chain was essential for Beryllium-specific T cell responses [24]. Thus, binding topologies of TCR to pMHC are not uniform among metal-specific TCRs because contact residues essential for their binding vary among metal ions. Pd-specific TCRs probably use germline-encoded CDRs and CDR3α but not CDR3β regions for the recognition of Pd-modified antigen. This intriguing finding may increase our understanding of antigen recognition by metal-specific TCR.

In conclusion, we have demonstrated that CD4+ Th1 cells infiltrated into the site of allergic inflammation in a murine metal allergy model, and estabilished the Pd-allergic specific T-cell clones. Infiltrated T cells used a limited TCR repertoire expressing AV18-1 and/or BV8-2. TCR clonotypes bearing AV18-1 had highly homologous CDR3 sequences among different mice, while BV8-2 exhibited diverse CDR3 sequences. These results suggest that restricted TCR AV18-1 with the CDR3 sequence (CAT-LYSGGS-NAKLTF) and broad TCR BV8-2 probably has the potential to specifically recognize Ag in Pd allergy. The direct cloning of TCR genes from local sites of inflammation using this model would be a powerful tool for understanding T-cell mediated immune disease in metal allergy, as well providing new insights into Ag recognition by Pd-specific TCR. 
